# Supplementary material for: Insights into Brochosome Distribution, Synthesis, and Novel Rapid-Release Mechanism in Maiestas dorsalis (Hemiptera: Cicadellidae)
Source: Insects. 2023 Aug 30;14(9):734. doi: 10.3390/insects14090734 (PMC10531587; doi:10.3390/insects14090734)
Supplement: Supplementary file 1 [file insects-14-00734-s001.zip › insects-2583573-supplementary.pdf]

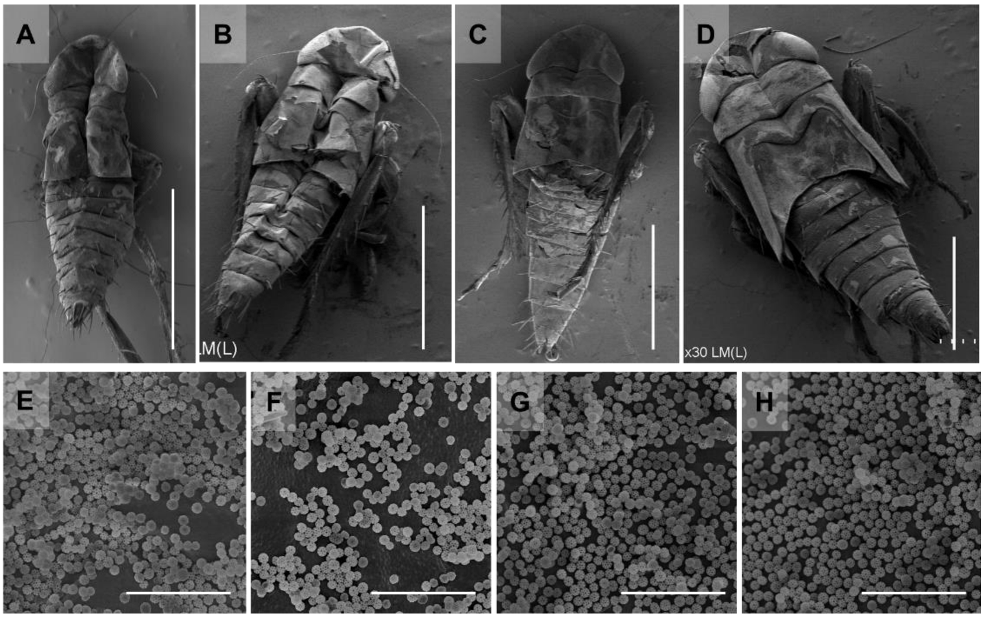

Figure S1. Distribution of brochosomes on the integument of leafhopper *M. dorsalis* at different developmental nymph stages.

Distribution of brochosomes on the integument of 2nd-instar nymphs (A and E), 3rd-instar nymphs (B and F),4th-instar nymphs (C and G), 5th-instar nymphs (D and H). Scale bars in A-D, 1 mm; E-H, 5 μm. All images are representative of at least three replicates.

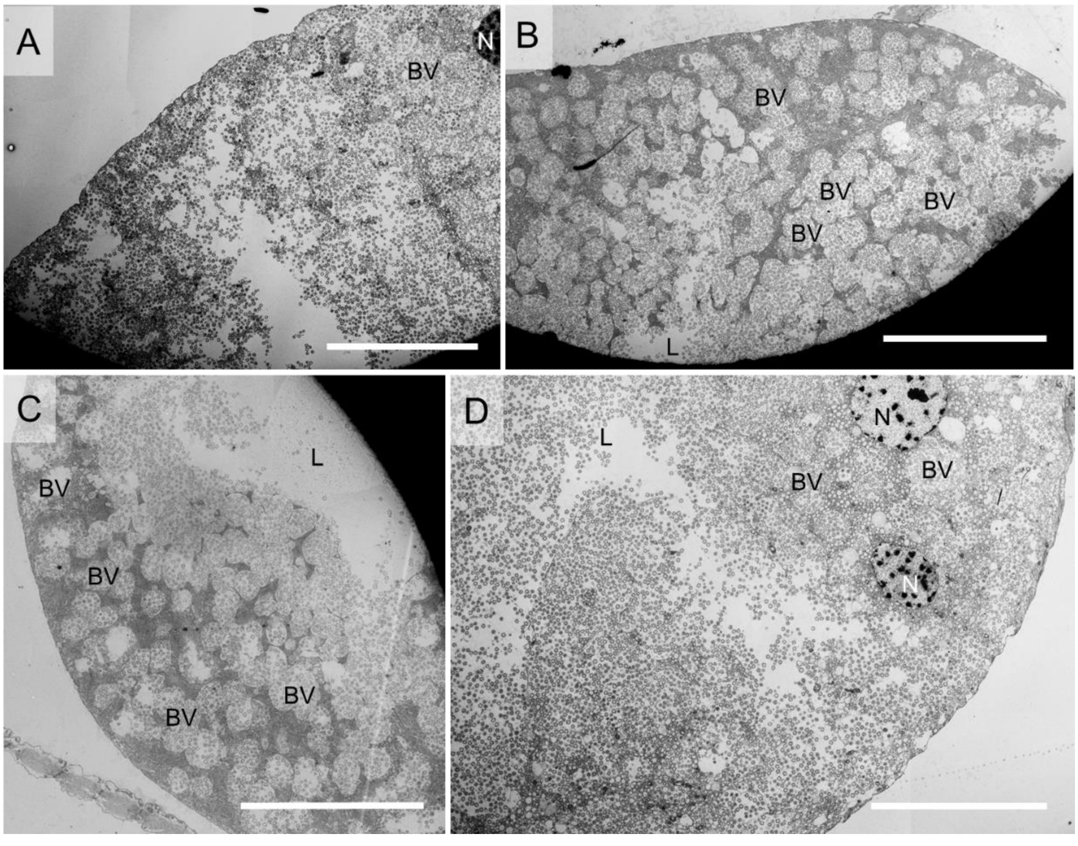

Figure S2. At 0 hours post-molt for 2nd to 5th instar nymphs, a substantial cavity formed by the fusion of multiple BV is observed in the epithelial cells of distal segments of the Malpighian tubules. (A) Second instar nymph. (B) Third instar nymph. (C) Fourth instar nymph. (D) Fifth instar nymph. BV, brochosomes-containing vesicles; L, lumen; N, nucleus. Scale bars in A-D, 20 μm. All images are representative of at least three replicates.
